# Advances in the Research and Application of Smart-Responsive Hydrogels in Disease Treatment

**DOI:** 10.3390/gels9080662

**Published:** 2023-08-17

**Authors:** Juan Cao, Ping Yuan, Bo Wu, Yeqi Liu, Cheng Hu

**Affiliations:** 1 School of Fashion and Design Art, Sichuan Normal University, Chengdu 610066, China; j.cao@sicnu.edu.cn; 2 School of Mechanical Engineering, Chengdu University, Chengdu 610106, China; evenyuan@126.com; 3 School of Mechanical Engineering, Sichuan University, Chengdu 610065, China; 743234238a@gmail.com (B.W.); 2022323020011@stu.scu.edu.cn (Y.L.); 4 National Engineering Research Center for Biomaterials, Sichuan University, Chengdu 610065, China

**Keywords:** smart-responsive, hydrogel, biomaterials, disease treatment

## Abstract

Smart-responsive hydrogels have been widely used in various fields, particularly in the biomedical field. Compared with traditional hydrogels, smart-responsive hydrogels not only facilitate the encapsulation and controlled release of drugs, active substances, and even cells but, more importantly, they enable the on-demand and controllable release of drugs and active substances at the disease site, significantly enhancing the efficacy of disease treatment. With the rapid advancement of biomaterials, smart-responsive hydrogels have received widespread attention, and a wide variety of smart-responsive hydrogels have been developed for the treatment of different diseases, thus presenting tremendous research prospects. This review summarizes the latest advancements in various smart-responsive hydrogels used for disease treatment. Additionally, some of the current shortcomings of smart-responsive hydrogels and the strategies to address them are discussed, as well as the future development directions and prospects of smart-responsive hydrogels.

## 1. Introduction

Hydrogels, as a type of hydrophilic polymer material, possess some characteristics of both solids and liquids. On the one hand, the three-dimensional crosslinked network of hydrogels gives them deformability and softness similar to elastic solids. On the other hand, their high water content allows them to exhibit liquid-like properties, such as permeability to various chemicals and biomolecules [1,2]. Additionally, thanks to the combination of the polymer network (i.e., solid) and water (i.e., liquid), hydrogels also demonstrate unique swelling behavior and responsive properties, making them promising in various fields such as industry [3], agriculture [4], and biomedical applications [5]. The limitations of traditional hydrogels, such as single functionality and insensitivity to environmental changes, have restricted their applications. To overcome these challenges, smart-responsive hydrogels have emerged, whose structure and properties are capable of responding to changes in external stimuli [6,7]. Over the past few decades, with the expanding boundaries of modern medicine and the increasing demand for precision treatment by patients, smart-responsive hydrogels have evolved significantly, greatly expanding their biomedical applications [8].

**Figure 1:**
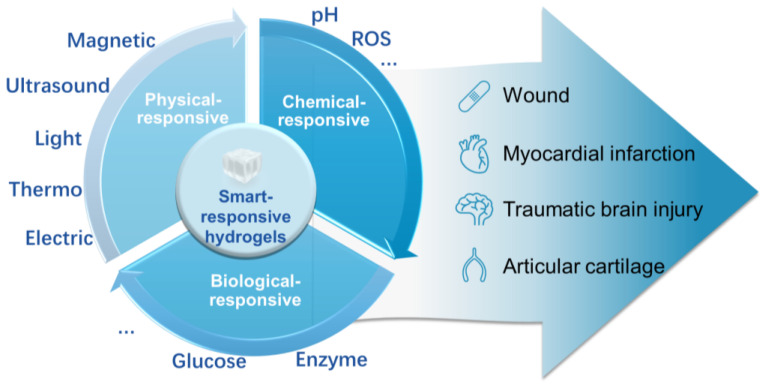
Classification and application of smart-responsive hydrogels in disease treatment.

Currently, smart-responsive hydrogels have emerged as advanced biomaterials for novel applications. By incorporating functional units or components, various types of smart hydrogels can respond to different physical (thermo-, light, electric field, magnetic field, etc.), chemical (pH, ions, biomolecules, etc.), or biological (enzyme or antigen reaction) stimuli. These responses induce specific conformational changes in polymer chains or alterations in the polymer network, leading to significant volume contraction/expansion, color changes, phase transitions, and more [9]. Different types of smart hydrogels exhibit various advantages, including high swelling ratios, flexibility, excellent biocompatibility, and similarity to human biological tissues [10]. Moreover, under specific stimuli, smart hydrogels can achieve programmable and controllable on-demand responses, greatly improving the diagnostic and therapeutic experiences for patients, and presenting broad prospects for clinical translation [11] In recent years, they have become one of the most promising materials in disease treatment (as shown in Figure 1).

In this review, we first comprehensively analyzed existing academic research to systematically discuss the classification and research progress of smart-responsive hydrogels. Subsequently, based on the diverse roles of smart-responsive hydrogels in disease treatment, we focused on the current applications of these hydrogels in wound repair, myocardial infarction repair, brain injury repair, and cartilage repair. Finally, we discussed the technical challenges and future development directions of smart-responsive hydrogels in biomedical applications, with a view to promoting further innovation and improvement of smart-responsive hydrogels in the field of disease treatment.

## 2. Types of Smart-Responsive Hydrogels

Smart-responsive hydrogels can be mainly categorized into three types, based on their response mechanisms: physical-, chemical-, and biological-responsive hydrogels [12,13]. Physical-responsive hydrogels mainly respond to changes in different signals in the physical environment, including temperature, light, electric fields, magnetic fields, ultrasound, etc. Chemical-responsive hydrogels, on the other hand, exhibit responses to changes in the chemical environment, such as pH and reactive oxygen species (ROS). Some hydrogels can also respond to biological molecules like enzymes and glucose, triggering biological or biochemical reactions, and these are referred to as biological-responsive hydrogels. Currently, there is extensive research on single-stimulus responsive hydrogels. However, in typical cases, the microenvironment of human disease sites is complex, and the application conditions for smart-responsive hydrogels may require responses to multiple stimuli simultaneously. Therefore, the development and application of dual- or even multiple-stimulus responsive hydrogels have attracted significant attention.

### 2.1. Physical-Responsive Hydrogels

#### 2.1.1. Thermo-Responsive Hydrogels

Thermo-responsive hydrogels, also known as temperature-responsive hydrogels, are one of the most extensively studied types within smart-responsive hydrogel systems. They are a class of hydrogels that undergo volume contraction or expansion in response to temperature changes. Typically, the polymer chains of these hydrogels contain a certain proportion of hydrophilic and hydrophobic groups. The phase transition of these hydrogels is achieved by reversible changes in hydrophilic/hydrophobic interactions within the internal network or through hydrogen bonding interactions [14,15,16]. When the external temperature increases or decreases to a critical value, even a slight temperature change can cause a significant volume change in the hydrogel, ranging up to several times, and this phenomenon is known as the volume phase transition behavior of the hydrogel [17]. Depending on the trend of swelling behavior with temperature changes, temperature-responsive hydrogels can be classified into two types: thermo-responsive expansion and thermo-responsive contraction (positive and negative temperature responses). Thermo-responsive expansion hydrogels (positive temperature response) have an upper critical solution temperature (UCST) [18]. These hydrogels tend to expand when the temperature exceeds the UCST and contract when the temperature decreases below the UCST. On the other hand, thermo-responsive contraction hydrogels (negative temperature response) have a lower critical solution temperature (LCST) [19]. They tend to shrink or collapse as the temperature increases above the LCST and expand when the temperature decreases below the LCST. Common thermo-responsive contraction hydrogels include amides, acrylates, ethers, etc., among which Poly(N-isopropylacrylamide) (PNIPAm) hydrogel is the most representative and widely used type [20]; its LCST is typically around 32 °C, close to the physiological temperature of the human body, making it promising for various biomedical applications [19,21].

#### 2.1.2. Light-Responsive Hydrogels

Light-responsive hydrogels are composed of a polymer network and light-sensitive groups as functional parts. Under appropriate light irradiation with specific intensity and wavelength, they can undergo physical/chemical property changes [22,23]. Light irradiation as a stimulus offers universality, practicality, and ease of use [24]. By adjusting parameters such as light intensity, wavelength, and exposure time, it can be precisely controlled remotely without the need for additional reagents [25,26,27]. Light-responsive hydrogels can be categorized into ultraviolet (UV)-responsive and visible light-responsive hydrogels, and, unlike UV light, visible light is readily available, safe, clean, cost-effective, and easy to manipulate [28,29,30]. There are three main mechanisms through which light-responsive hydrogels exhibit characteristic changes under light stimulation: First, by introducing photosensitive molecules into the hydrogel, the light-sensitive groups undergo changes in their chemical properties upon absorbing a certain amount of photons, triggering the hydrogel’s phase transition mechanism and resulting in different responsive properties, such as volume and color changes [31]. Second, photosensitive substances in the hydrogel can absorb light energy and convert it into heat, raising the hydrogel’s temperature to reach the phase transition temperature, leading to a change in its volume, density, and other physical properties [9]. The third mechanism involves the decomposition of photosensitive molecules within the hydrogel under light exposure, leading to reactions or changes in the hydrogel network, altering the osmotic pressure, and causing swelling or shrinking of the hydrogel [25].

#### 2.1.3. Electric/Magnetic-Responsive Hydrogels

Hydrogels that respond to electric and magnetic fields by changing their properties in response to slight variations in electric current or external magnetic fields are known as electric/magnetic-responsive hydrogels. In electric-responsive hydrogels, when the surrounding electric field changes, ions within the hydrogel move along specific directions under the electric field, leading to a difference in ion concentration on both sides, which causes a variation in osmotic pressure, resulting in changes in the hydrogel’s volume [32]. The extent of change in electric-responsive hydrogels depends on factors such as contact with electrodes, electric field intensity, and electrolyte content in the solution. Although the response of these hydrogels can be precisely controlled by applying an external electric field, their drawback is a relatively slow response speed [33]. Magnetic-responsive hydrogels, on the other hand, are a class of environmentally sensitive hydrogels that exhibit response characteristics to magnetic fields [34]. These hydrogels can use an external magnetic field to achieve rapid and effective control of their motion and direction and have garnered considerable attention. By incorporating magnetic particles into different hydrogel matrices in various ways, a wide range of magnetic-responsive hydrogels with different properties and functionalities can be obtained [35]. The commonly used magnetic particles are γ-Fe_2_O_3_, Fe_3_O_4_, CoFe_2_O_4_, etc. [36]; the type, concentration, size, and distribution of these magnetic particles affect the response of the hydrogels [37]. When a certain magnetic field is applied externally, the aggregation of magnetic particles within the hydrogel causes the network structure to shrink. When the magnetic field is removed, the hydrogel network molecules can freely diffuse and return to their original expanded state. Compared to other types of responsive hydrogels, magnetic-responsive hydrogels possess the characteristics of rapid response, ease of manipulation, and the ability to achieve precise control, making them highly promising for various applications.

#### 2.1.4. Ultrasound-Responsive Hydrogels

Ultrasound is typically a simple and convenient physical stimulus that has been used as a non-invasive external stimulus in the design and preparation of smart hydrogels [38]. Currently, the most common ultrasound-responsive hydrogels involve the combination of hydrogels with ultrasound imaging technology. This can be achieved by using intrinsically imaging-compatible polymers to prepare hydrogels or by adding contrast agents to the hydrogel to enhance the ultrasound diagnostic echo signals and accuracy, providing a versatile and powerful platform for biomedical imaging [39]. The response mechanisms of ultrasound-responsive hydrogels to ultrasound stimuli can be categorized into three types [40,41]: The first one involves utilizing the ultrasound cavitation effect to disrupt the crosslinking structure of the hydrogel. Under ultrasound irradiation, the pressure and shock waves generated by the ultrasound cavitation can break the polymer chains of the hydrogel. The second mechanism is that ultrasound stimulation can be absorbed by the hydrogel and converted into heat, inducing localized hyperthermia. This hyperthermia can trigger phase transitions in hydrogels, enabling them to respond as required. The third is that ultrasound generates an acoustic streaming effect during propagation, which can push drug molecules carried by the hydrogel to move within pores. By setting appropriate ultrasound parameters, such as frequency, acoustic pressure, and irradiation time, various types of ultrasound-responsive hydrogels can be designed and applied in different fields [42,43].

### 2.2. Chemical-Responsive Hydrogels

#### 2.2.1. pH-Responsive Hydrogels

pH-responsive hydrogels are composed of polymer main chains with ionizable functional groups that respond to changes in the surrounding solution’s pH, leading to alterations in the electrostatic interactions between the internal polymer networks, resulting in a phase transition [44,45,46]. The pH value determines the charged state of the ionizable groups on the hydrogel polymer chain, such as carboxyl or amino groups, altering their charge density and causing a difference in osmotic pressure between the inside and outside of the hydrogel [47,48]. Depending on the nature of the side groups within the internal polymer network, pH-responsive hydrogels can be categorized as anionic, cationic, or amphoteric.

In anionic hydrogels, the ionizable groups are typically weak acidic groups on the polymer chain. When the pH is lower than the pKa (the acid dissociation constant), the carboxyl groups are only partially ionized, resulting in weak electrostatic repulsion between the polymer chains, causing the hydrogel to shrink. Conversely, when the pH is higher than the pKa, the ionization of the carboxyl groups increases significantly, leading to stronger electrostatic repulsion between the polymer chains, causing the hydrogel to swell [49]. Cationic hydrogels operate on a similar swelling mechanism to anionic hydrogels but with opposite trends. In cationic hydrogels, the ionizable functional groups are typically amine groups on the polymer chain. The swelling behavior of both anionic and cationic hydrogels depends on the number and ionization degree of the charged groups on the polymer chains, with a higher number and greater ionization degree of ionizable groups leading to more significant swelling [50]. Amphoteric hydrogels, containing both amine and carboxyl groups on their polymer chains, both of which can change their charge state, exhibit a more complex expansion and contraction behavior [51]. The swelling behavior of amphoteric hydrogels is influenced by the type, quantity, and position of the anionic and cationic groups along the polymer chains [52,53]. In recent years, pH-responsive hydrogels based on Schiff base chemistry have also gained significant attention and application. By forming dynamic covalent bonds (Schiff base) between aldehyde and amine groups, hydrogels can be rapidly formed within a short period. The Schiff base can be hydrolyzed under acidic conditions, providing the hydrogel with fast and reversible pH-responsive characteristics. This hydrogel demonstrates excellent stability under neutral conditions but undergoes a gel-to-solution transition as the environment shifts towards weak acidity [54,55].

#### 2.2.2. ROS-Responsive Hydrogels

ROS-responsive hydrogels can perceive the levels of ROS in the environment and respond through changes in solubility or chemical bond degradation [56]. ROS refer to various chemically derived oxygen molecules [57], including free radicals such as superoxide (O_2_), hydroxyl (OH), peroxyl (RO_2_), and alkoxyl (RO), as well as non-radicals such as hypochlorous acid (HOCl), ozone (O_3_), singlet oxygen (^1^O_2_), and hydrogen peroxide (H_2_O_2_). ROS at appropriate concentrations play critical roles in various biological functions in living organisms, such as regulating cell signaling, hormone production, protein function modulation, or mediating inflammation [58,59,60]. However, excessively high ROS levels are closely associated with the occurrence and progression of various diseases [61,62]. Therefore, ROS-responsive hydrogels are ideal candidates for developing stimuli-responsive biomaterials for targeted therapy [63]. The design principle of ROS-responsive hydrogels is based on the interaction between ROS and specific functional groups within the material. One common strategy is to introduce monomers containing oxygen-sensitive functional groups into the hydrogel network, such as thiol (-SH) or selenol (-SeH) groups [64]. These functional groups can undergo oxidation reactions with ROS, leading to the formation of oxidative by-products, which cause the hydrogel network to break or deform. When the ROS concentration decreases or disappears, the oxidative by-products can be reduced, thereby restoring the structure and functionality of the hydrogel. Boronic acid and its derivatives are a class of non-natural, synthetically derived diol receptors that can undergo reversible reactions with polyhydroxy compounds in an aqueous solution, forming covalent complexes. Due to their ROS responsiveness, these covalent complexes have found wide applications in tissue engineering and drug delivery fields [65]. In recent years, various ROS-responsive hydrogel systems based on boronic acid crosslinking have been reported, including sodium alginate, hyaluronic acid, cellulose, chitosan, gelatin, etc. [66,67]. The sensitivity of ROS-responsive functional groups under different conditions depends on various factors such as the type of ROS, polymer structure, material form, and exposure time [68]. This property enables them to self-assemble into micellar nanoparticles or hydrogels in aqueous solutions, primarily used as drug carriers for localized treatment of damaged body regions characterized by high ROS generation [58].

### 2.3. Biological-Responsive Hydrogels

#### 2.3.1. Enzyme-Responsive Hydrogels

Enzymes are among the most essential components in our bodies, participating in virtually all biological processes. Enzymes are ubiquitous in every part of the body and exhibit high substrate specificity [69], thus drawing widespread attention. Enzyme-responsive hydrogels are chemically crosslinked polymer networks prepared using nature-sensitive natural polymers (e.g., fibrinogen, collagen, gelatin, and hyaluronic acid) or biocompatible materials with enzyme-sensitive moieties [70,71], whose fundamental response mechanism involves the interaction of enzymes with specific chemical bonds in the hydrogel, which can trigger chemical reactions, thereby altering the physical and chemical properties of the hydrogel, such as shape, stiffness, porosity, or solubility. Various types of enzymes are used in enzyme-responsive hydrogel systems [72], including proteases, kinases, phosphatases, and (d) nucleases. To ensure the proper functioning of enzyme-responsive hydrogels, the active enzyme interactions with the hydrogel should be maintained, allowing the enzymes to move towards their substrates anchored within the hydrogel through diffusion kinetics. However, not all enzymes are capable of cleaving and degrading chemical bonds. For instance, transglutaminase is one of the numerous enzymes that form isopeptide bonds between peptides/proteins. By applying these characteristics to enzyme-responsive hydrogel systems, the formation of internal crosslinks can significantly enhance their structural strength [73]. Similarly, different types of enzymes and enzyme-responsive hydrogels can interact dynamically, leading to the formation and deformation of hydrogel structures, enabling a wide range of applications in the biomedical research field, such as drug delivery, enzyme sensing, and therapies [74,75,76,77].

#### 2.3.2. Glucose-Responsive Hydrogels

Glucose-responsive hydrogels can alter their sol–gel behavior in response to changes in the external glucose concentration. The commonly used glucose-responsive hydrogels include concanavalin A (Con A) hydrogel, glucose oxidase hydrogel, and phenylboronic acid (PBA) hydrogels [8,78,79]. Glucose-responsive hydrogels can be applied in various forms, such as microgels, nanogels, vesicles, micelles, cells, microneedle patches, and mesoporous nanoparticles [80]. Each form of glucose-responsive hydrogel can undergo changes in binding capacity or system structure by sensing variations in glucose concentration. These changes may involve substrate expansion/contraction, material dissolution, pore size alteration, and/or carrier degradation [81]. Glucose-responsive hydrogels have found significant applications in the biomedical field, including biosensors for detecting glucose levels in body fluids of diabetes patients and as medical dressings for diabetic wound care, among others [82,83].

### 2.4. Dual- or Multiple-Responsive Hydrogels

As mentioned above, hydrogels can respond to various stimuli according to specific needs and applications. Usually, smart-responsive hydrogels are faced with complex application environments where multiple stimuli and signals exist. Therefore, to enhance the functionality and applicability of intelligent hydrogels, the development and application of dual- or even multiple-stimulus-responsive hydrogels have garnered widespread attention. One of the simplest approaches to developing multiple-response hydrogels is to combine individual stimuli into existing composite hydrogel systems [84]. For example, some thermo-responsive hydrogels can be modified with appropriate functional groups to respond to light stimulation [85]. Similarly, by incorporating conductive elements (such as polymers and metal nanoparticles), existing hydrogels can be converted into electric-responsive hydrogels [86]. The introduction of magnetic colloidal particles into the hydrogel matrix allows the entire system to form various structures of magnetic-responsive hydrogels under external magnetic field control [87]. By combining magnetic fields with iron oxide nanoparticles, not only can induced electric fields be generated within these particles, but also phase transitions in the thermo-responsive polymer matrix can be induced. Consequently, by screening particles and polymer matrices, triple-responsive hydrogels that respond to magnetic, electric, and temperature stimuli can be prepared [88].

## 3. Applications of Smart-Responsive Hydrogels in Disease Treatment

In recent years, the applications of these smart-responsive hydrogels have attracted significant attention and have made tremendous progress. Various types of smart hydrogels, by responding to different chemical, physical, or biological stimuli, either singularly or multiply, have not only exhibited advantages such as high swelling ratio, flexibility, excellent biocompatibility, and similarity to human biological tissues, but they have also achieved programmable and controllable on-demand responsiveness. This has greatly enhanced the patients’ treatment experience and holds vast potential for clinical translation. These hydrogels have emerged as one of the most promising materials, particularly in the areas of wound repair, myocardial infarction treatment, traumatic brain injury treatment, and articular cartilage repair and regeneration, as shown in Table 1. Smart-responsive hydrogels have evolved from the foundation of hydrogel technology, and their clinical translation and commercialization are currently in the research stage. Building upon years of development, hydrogel products suitable for a variety of indications have entered clinical trials or have received clinical approval, as shown in Table 2.

### 3.1. Wound Repair

**Figure 2:**
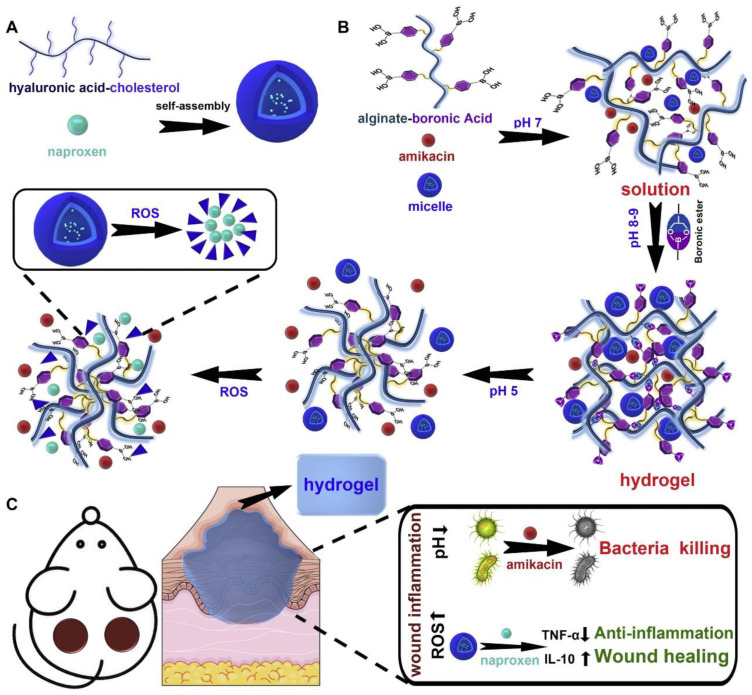
(**A**) Preparation scheme of the micelle. (**B**) Schematic illustration of the formation of hydrogels. (**C**) Schematic illustration of the mechanisms that control anti-bacterial and wound healing. Copyright permission from Hu et al. [90], Journal of Controlled Release, 2020.

Wound healing is a complex dynamic process consisting of four consecutive and overlapping stages: hemostasis, inflammation, cell proliferation, and tissue remodeling [109]. Improper treatment can exacerbate wound infections and, in some cases, even threaten life [2,110]. Hydrogels offer unique advantages in wound repair due to their excellent hydrophilicity, biocompatibility, and three-dimensional porous structures, resembling the extracellular matrix [111]. Smart-responsive hydrogels, through flexible structural modifications, the integration of different functional components, and the loading of bioactive substances, have been widely applied in wound repair research and clinical practice, as they can promptly respond to changes in the wound microenvironment [112]. Temperature and pH are important physicochemical factors in the human body, and stimulus-responsive hydrogels, designed based on variations in surface pH and temperature, can effectively accelerate wound healing. For instance, Hanif et al. [89] prepared a pH and temperature dual-stimulus-responsive hydrogel by crosslinking N-isopropyl acrylamide with acrylic acid and loading ultrasmall silver nanoparticles (AgNPs). Under an acidic pH (pH < 5), Ag^+^ release was restricted, whereas it significantly promoted Ag^+^ release (>90% release) at an alkaline pH (pH > 7.4). The hydrogel exhibited weak bactericidal activity at pH 4 or 5.5 and could eliminate 95% of pathogens at pH 7.4 and 10, effectively clearing Staphylococcus aureus infection and accelerating wound healing. Accumulation of ROS generated from wound or bacterial infections can impair cell proliferation, disrupt angiogenesis, and hinder wound healing [113]. Hu et.al. [90] designed an intelligent injectable hydrogel with self-healing properties, incorporating boronic acid groups onto alginate polymer chains to create a high ROS-responsive hydrogel. Simultaneously, by assembling micelles of the antibiotic amikacin (AM) and anti-inflammatory agent naphthalene propylamine (Nap) into the hydrogel, it gained antibacterial and anti-inflammatory capabilities. This hydrogel formulation not only maintained the structural integrity and excellent rheological properties of hydrogels but also provided controlled drug release at inflammatory sites, effectively promoting wound healing (Figure 2). In another study, they developed a pH/ROS dual-responsive hydrogel, by incorporating sodium diclofenac (DS) and mannosylated insulin-chitosan micelles (MIC@MF) into different spatial locations of the dual-responsive hydrogel, to construct a spatiotemporally controllable drug delivery system [91]. In vitro and in vivo experiments confirmed that DS&MIC@MF hydrogel exhibited good biocompatibility and inherent antibacterial and antioxidant abilities. Rapid DS release exhibited effective antibacterial and anti-inflammatory properties, while sustained MF release promoted blood vessel regeneration, collectively accelerating wound healing, and providing a promising therapeutic strategy for chronic diabetic wound healing. In addition, the patent [114] reported an ROS-responsive microneedle patch for the treatment of acne or other infectious skin wounds. Carbon dioxide (CO_2_) has been recognized as one of the most effective methods to improve microcirculation and benefit wound healing [115]. Recently, Ge et al. [92] used thermo-responsive Pluronic F127 block copolymers and amino-functionalized carbon nanoparticles (CNPs) to prepare a near-infrared light-responsive hydrogel for on-demand and localized delivery of CO_2_ to treat bacterial-infected wounds. Under near-infrared irradiation, CNPs converted light into heat, triggering the decomposition of bicarbonate to produce CO_2_. Thus, this photosensitive hydrogel could release CO_2_ at the local wound site, accelerating wound healing by improving local microcirculation and increasing tissue oxygen concentration. Currently, wound healing of deep tissue injuries remains challenging. Yang et al. [93] designed an intelligent hydrogel drug delivery system based on a novel two-dimensional layered nanomaterial (MXene) possessing light and magnetic responsiveness and controllable drug delivery capabilities for deep wound treatment. The hydrogel comprised MXene-encapsulated magnetic colloids and a dual-network hydrogel of poly(N-isopropyl acrylamide) and alginate, loaded with silver nanoparticles (AgNPs). Under near-infrared and alternating magnetic field (AMF) conditions, the temperature of the system rapidly increased, allowing the controlled release of AgNPs. This hydrogel demonstrated excellent cell compatibility and biocompatibility, reduced drug toxicity, and promoted wound healing.

### 3.2. Myocardial Infarction Treatment

**Figure 3:**
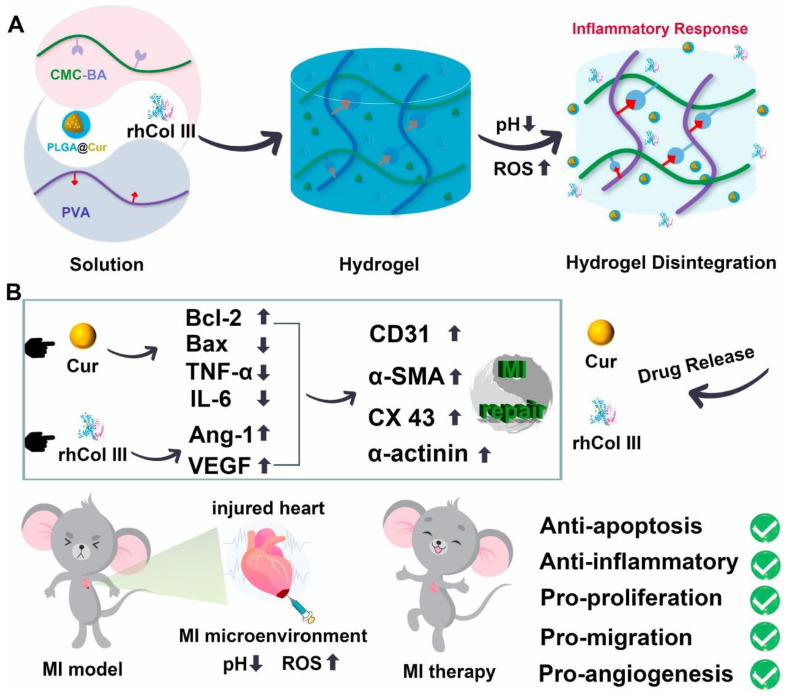
(**A**) Preparation process and drug release mechanism of MI-responsive hydrogels. (**B**) Molecular mechanisms of MI-responsive hydrogels in MI therapy. Copyright permission from Hu et al. [95], Biomaterials, 2022.

Myocardial infarction (MI) is a severe cardiovascular disease, and after infarction, the myocardial microenvironment undergoes various changes, including fluctuations in matrix metalloproteinases (MMPs), ROS, and inflammatory factors [116]. Smart-response hydrogels have been widely applied in MI treatment, often showing better efficacy than traditional inert hydrogels. These dynamic hydrogels can change in size and shape through the degradation of their crosslinked networks, enabling precise and controlled drug release at the appropriate time and location, achieved through external stimuli such as pH, temperature, light, electric fields, and magnetic fields. The accumulation of a large amount of ROS in the infarcted myocardium after MI can cause the death of myocardial cells in non-infarcted areas, leading to additional cardiac injury and an increased infarct area [117]. To reverse the adverse microenvironment after MI, Gao et al. [94] developed a ROS-responsive hydrogel by combining ROS-cleavable hyperbranched polyamide (HBPAK) with methacrylate hyaluronic acid (HA-MA). HBPAK was synthesized via Michael’s addition reaction between poly (ethylene glycol) diacrylate with a molecular weight of 575 (PEGDA575) and thiol-ketone aldimine diethylamine. The ROS responsiveness of the hydrogel could be adjusted by changing the HBPAK concentration, making it significantly meaningful for improving the MI microenvironment. Recently, Hu et al. [95] synthesized a microenvironment-responsive multifunctional hydrogel loaded with anti-inflammatory nanoparticles and customized recombinant type III humanized collagen (rhCol III) for repairing damaged myocardial tissue. This hydrogel could respond to acidic and high ROS environments at the heart failure site, selectively releasing the anti-inflammatory drug curcumin (Cur) and rhCol III. The Cur exhibited excellent antioxidant and anti-inflammatory properties, effectively reducing ROS levels and cell apoptosis, while the customized rhCol III promoted cell proliferation, migration, and angiogenesis. The experimental results demonstrated its enormous potential in accelerating damaged heart repair, opening up new directions for heart failure treatment (Figure 3). Due to the various physiological functions of nitric oxide (NO), delivering exogenous NO to the infarcted area has been shown to be an effective strategy for treating MI [118]. Hao et al. [96] reported a boronate-protected chitosan-modified bisnitroimidazole (CS-B-NO) injectable hydrogel that could respond to ROS stimuli to release NO, thereby regulating the ROS/NO imbalance after ischemia/reperfusion (I/R) injury. Additionally, in a myocardial I/R injury mouse model, CS-B-NO administration effectively alleviated cardiac injury and adverse cardiac remodeling compared to a hydrogel that only released NO, promoting cardiac repair and improving cardiac function. Conductive polymers such as polyaniline, PEDOT: PSS, polypyrrole, and their combinations with other polymers have been used to synthesize electrically responsive hydrogels [97,98]. These smart hydrogels help maintain/improve heart rhythm, thus enhancing cardiac contraction, while also enhancing the adhesion of myocardial cells. Similarly, hydrogels containing betaine carboxylate zwitterionic groups can also respond to electrical pulses and improve heart function, reducing fibrotic areas and accelerating the recovery of infarcted heart tissue [99]. Injectable hydrogels carrying therapeutic factors to regulate the infarcted immune environment have also shown great potential in the treatment of MI. Chen et al. [100] prepared an MMP2/9 (matrix metalloproteinase)-responsive hydrogel system (MPGC4) consisting of four-arm polyethylene glycol (PEG) hydrogel and IL-4 plasmid DNA complexed with carbon dots (CDots) gene nanocarrier (CTL4), aiming to modulate the MI immune microenvironment for MI treatment.

### 3.3. Traumatic Brain Injury Treatment

**Figure 4:**
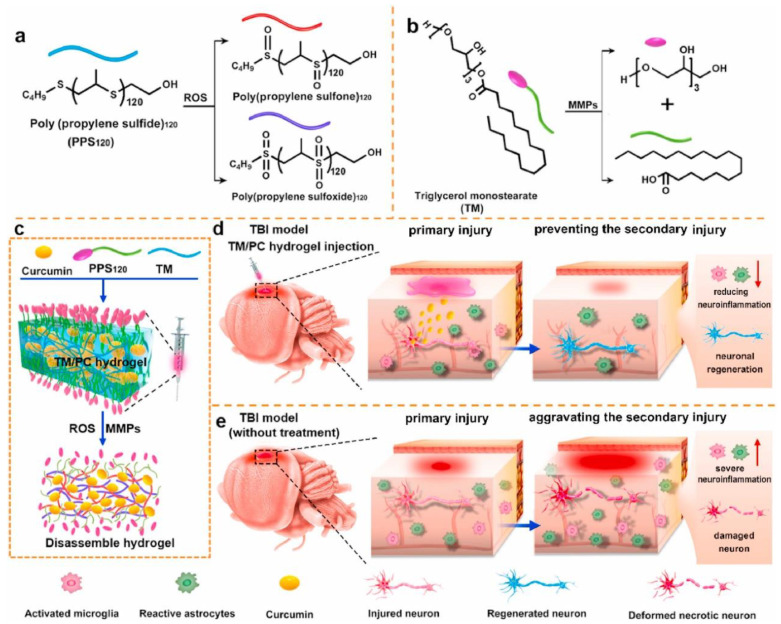
Formation and mechanism of the TM/PC hydrogel in TBI. (**a**) PPS120 switched from a hydrophobic polymer to the more hydrophilic poly (propylene sulfone)120 and poly (propylene sulfoxide)120 in the ROS environment. (**b**) TM could be degraded by MMPs. (**c**) Schematic illustration of TM/PC hydrogel preparation procedures and degradation process under MMP and ROS environment. (**d**) In situ injection of TM/PC hydrogels within the post-surgery TBI. (**e**) Without hydrogel treatment, neuronal death and severe neuroinflammation were observed, and the secondary injury was aggravated. Copyright permission from Qian et al. [101], Biomaterials, 2021.

Traumatic Brain Injury (TBI) is a common neurosurgical condition that can result in neural functional impairment, cell death, and delayed neuroinflammation, among other consequences. The repair and treatment of brain injuries pose a major global challenge in clinical medicine. Excessive ROS generated during TBI can exacerbate secondary injuries and lead to disability and death. Smart hydrogel materials, mimicking the extracellular matrix (ECM) characteristics of brain cells, offer certain advantages in promoting stem cell adhesion, proliferation, and differentiation, providing a clinical advantage in treating secondary injuries. Qian et al. [101] developed a ROS-responsive hydrogel by encapsulating hydrophobic polysulfide acrylate 120 (PPS120) and Cur, which can react with ROS, within the hydrophobic core of a trimethylolpropane triglycidyl ether (TM) hydrogel. The hydrogel can be degraded by matrix metalloproteinases (MMPs), forming an MMP-responsive TM/PC hydrogel that can regulate the ROS microenvironment of brain injury and facilitate brain injury repair. Brain injury results in the loss of neuronal function and ECM damage (Figure 4). Adak et al. [102] designed a sulfonic acid-functionalized injectable biocompatible peptide hydrogel, which not only simulates ECM to support damaged neurons but also responds to matrix metalloproteinase 9 (MMP9) to release neurotrophic factors at the brain injury site. This hydrogel promotes the synaptic growth of primary neurons and mitigates the neurotoxic effects induced by anti-nerve growth factor (Anti-NGF), demonstrating great potential in promoting neural regeneration and facilitating rapid brain recovery after injury. Increasing evidence suggests that stem cell-based neural repair therapies hold great promise for stroke patients with diabetes [119]. Yao et al. [103] developed a novel thermosensitive hydrogel (CS-HEC-HA/GP) based on chitosan, hydroxyethyl cellulose, hyaluronic acid, and β-glycerophosphate. Compared to CS/GP or CS-HEC/GP hydrogels, the CS-HEC-HA/GP hydrogel exhibited a faster gelation process and better biocompatibility with human umbilical cord mesenchymal stem cells (hUC-MSCs). Furthermore, the hUC-MSC-loaded CS-HEC-HA/GP hydrogel enhanced the viability and migration of encapsulated hUC-MSCs, leading to increased survival and proliferation of endogenous neural cells through the secretion of neurotrophic factors and inhibition of apoptosis. This process accelerated the structural remodeling of the rat brain and the recovery of neural function after TBI. Jiang et al. [104] reported a glucose/reactive ROS-responsive hydrogel capable of encapsulating extracellular vesicles (EVs) and controllably releasing them in response to the brain microenvironment after stroke in type 2 diabetic mice. The EV-hydrogel was developed by crosslinking hyaluronic acid and polyethylene glycol, modified with phenylboronic acid, and fused with EVs derived from neural stem cells. It exhibited excellent injectability, biocompatibility, and self-healing ability, promoting angiogenesis and improving neurological behavior recovery in mice, providing a safe and effective treatment for stroke patients with diabetes.

### 3.4. Articular Cartilage Repair and Regeneration

**Figure 5:**
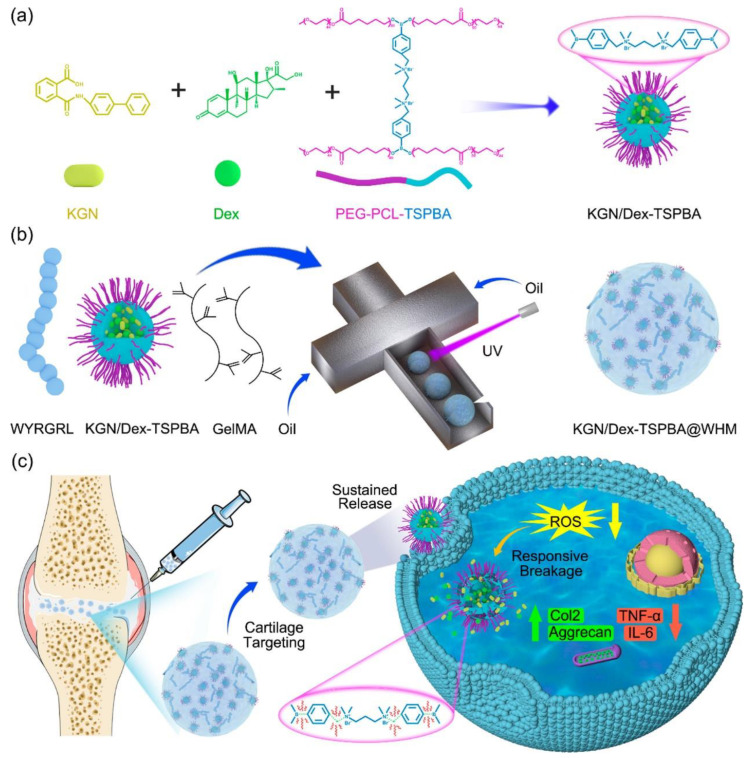
Schematic illustration of nanoparticle assembly, hybrid HM fabrication, and OA therapy. (**a**) The fabrication of the KGN/Dex-TSPBA nanoparticles. (**b**) The fabrication of KGN/Dex-TSPBA@WHMs. (**c**) The mechanism of KGN/Dex-TSPBA@WHMs in the treatment of OA. Copyright permission from Yu et al. [105] ACS Applied Materials & Interfaces, 2022.

Diseases of cartilage caused by injury, trauma, or inflammatory disease, such as osteoarthritis, are common orthopedic conditions that usually result in defective articular cartilage, causing significant physical and psychological burdens on the patient [120,121]. Additionally, due to the avascular nature of cartilage, its self-regeneration capability is limited. Consequently, articular cartilage regeneration and repair remain challenging issues. The failure of conventional treatments has prompted researchers to search for new alternatives, among which the application of smart-response hydrogels undoubtedly represents an effective and promising approach for articular cartilage repair and regeneration [122]. Excessive ROS can induce chondrocyte apoptosis, activate the production of inflammatory factors and MMPs, and lead to the degradation of joint cartilage ECM [68]. Therefore, researchers have designed a series of ROS-responsive drug-releasing hydrogels for treatment. Yu et al. [105] used microfluidic technology to anchor cartilage-targeting peptides and ROS-responsive nanoparticles in a hydrogel matrix, designing and synthesizing ROS-responsive biomimetic cartilage-targeting hydrogel microspheres, and providing a new therapeutic strategy for cartilage repair (Figure 5). Wu et al. [123] prepared a novel composite scaffold with excellent mechanical properties and anti-inflammatory function by filling a ROS-responsive hydrogel into a PLGA scaffold with radially oriented pores. In vitro cell experiments demonstrated that the composite scaffold effectively supported the survival and proliferation of bone marrow mesenchymal stem cells (MSCs) and protected cells under oxidative stimulation, maintaining normal ROS levels. In an animal model of rabbit cartilage defects, the composite scaffold effectively suppressed joint inflammation and promoted the regeneration of hyaline cartilage, providing a basis for designing cartilage repair scaffolds. MSCs possess immunomodulatory, anti-inflammatory, and tissue and organ regeneration and repair functions, making them particularly effective in promoting cartilage regeneration. Zeng et al. [106] developed a “mussel-inspired” multi-responsive hydrogel that can co-deliver exosomes (Exos) and icariin (ICA) for the synergistic and efficient treatment of osteoarthritis. The successfully constructed ICA-loaded exosome (ICA@Exos) and ICA@Exos-gel played a synergistic and enhanced effect in promoting cell proliferation, migration, and inhibiting MMP secretion, and significantly prolonged the retention of exosomes in vivo, forming a reliable drug depot at the joint and playing a significant role in cartilage repair. Many exogenous stimuli have also been applied in smart-responsive hydrogels for cartilage repair and regeneration. For example, ultrasound, due to its high controllability, non-invasiveness, and high tissue permeability, can serve as an external mechanical force stimulus in smart drug delivery systems for joint cartilage repair [124]. Yuan et al. [107] prepared a carboxymethyl chitosan/oxidized chondroitin sulfate (CMC-OCS) hydrogel containing ultrasound-responsive microspheres through a Schiff base crosslinking reaction. The ultrasound-responsive microspheres (PLGA MPs@KGN) were prepared using a rapid membrane emulsification method and loaded with a potent inducer, kartogenin (KGN), which promoted the differentiation of MSCs into chondrocytes. The composite hydrogel demonstrated excellent cartilage repair effects in vitro and in vivo. Lv et al. [108] reported a novel injectable smart thermo-responsive hydrogel (CSP-LB), mainly composed of MgFe-LDH nanosheets loaded with BMP-2 and PDGF-BB, and a chitosan/fibroin (CS) matrix. The CSP-LB hydrogel achieved a burst release of PDGF-BB and sustained release of BMP-2, overcoming the limitations of existing bone-repair hydrogels and effectively meeting the controlled release requirements of different growth factors during bone defect treatment, showing excellent therapeutic efficacy.

## 4. Conclusions and Future Perspectives

With the rapid development of biomaterials and tissue engineering, smart-response hydrogels, as innovative materials, have shown tremendous potential in the field of disease treatment. An increasing number of smart hydrogels have been reported for the treatment of various diseases, achieving favorable therapeutic outcomes. These hydrogels possess the ability to respond to specific environmental signals, such as changes in temperature, pH, light, ROS, enzymes, and glucose. This unique characteristic enables smart hydrogels to deliver drugs or active substances in a controlled and targeted responsive manner at the site of the disease after incorporating different therapeutic agents, leading to better disease treatment while reducing drug administration frequency and side effects. 

Although smart-responsive hydrogels have made remarkable progress in disease treatment, so far, no smart hydrogel has obtained clinical approval. It is foreseeable that smart-responsive hydrogels still face significant challenges in practical application and clinical translation, mainly in the following three aspects: The first key challenge is the insufficient responsiveness of current hydrogels. External stimuli often require higher intensities of stimulation (such as electrical, magnetic, ultrasound, and light) to trigger a response, which may also lead to potential harm to the human body. For internal stimuli, if the levels of enzymes, reactive oxygen species, glucose, and other substances in the body are low, the response cannot be triggered. The second challenge lies in the overly complex design of current smart hydrogel systems. On the one hand, this complexity hinders the guarantee of biocompatibility and safety of smart-responsive hydrogels. On the other hand, it results in complex manufacturing processes and high costs, making it difficult to achieve large-scale production. The third key challenge is how to precisely control the release of drugs or active substances from smart-responsive hydrogels at disease sites. Therefore, further research is needed to explore more sensitive crosslinking bonds to improve the responsiveness of hydrogels, which is also the most important point for clinical translation and practical application. Additionally, it is necessary to produce a sufficient quantity of materials at a feasible cost during manufacturing, ensuring their quality and consistency. Consideration should also be given to factors such as drug stability, modulation of release rate, and temporal and spatial distribution of release, to achieve reliable controlled release in clinical applications.

Currently, a large number of researchers are making efforts to overcome the aforementioned challenges for the clinical translation and commercial application of smart-responsive hydrogels. With the advancement of precision medicine, smart-responsive hydrogels will undoubtedly play an increasingly important role. Smart-responsive hydrogels have the ability to dynamically adjust according to different physiological conditions and stimuli, providing personalized and precise treatment strategies. Their unique responsiveness allows for controlled on-demand drug release, thereby optimizing treatment effects while minimizing potential side effects. This adaptability also extends to the fields of diagnostics and monitoring, enabling high sensitivity and selectivity in biological detection, aiding in accurate early disease diagnosis and monitoring, and providing earlier and more effective treatment for patients. In conclusion, although smart-responsive hydrogels still face some challenges in practical applications, with ongoing technological advancements and research progress, they are poised to fundamentally revolutionize the approach to treating and managing diseases in the future. This transformation will enhance patients’ well-being and quality of life, contributing positively to human life, health, and scientific advancements.

## Figures and Tables

**Table 1:** Application of Polymer-Based Stimuli-Responsive Hydrogels in Disease Treatment.

Polymer Sources	Responsive Property	Synthetic Method	Applications	References
N-isopropylacrylamide with acrylic acid	pH/temperature response	Free-radical polymerization	Wound repair	[89]
Sodium alginate	ROS response	Phenyl boronic ester bonds		[90]
Oxidized dextran and ε-polylysine	pH/ROS response	Schiff base linkages and boronic ester bonds		[91]
Pluronic F127	Light response	Hydrophobic-hydrophilic interactions		[92]
Poly(N-isopropyl acrylamide) and alginate	Light/ magnetic response	Covalently crosslinked and ionically crosslinked		[93]
Hyaluronic acid	ROS response	Michael addition reaction	Myocardial infarction treatment	[94]
Carboxymethyl cellulose and Poly vinyl alcohol	pH/ROS response	Phenyl boronic ester bonds		[95]
Chitosan	ROS response	Boronate ester groups		[96]
Gelatin	Electric response	EDC/NHS crosslinking		[97,98]
Poly(carboxybetaine methacrylate)	Electric response	Michael addition reaction		[99]
Poly(ethylene glycol)	Enzyme response	Michael addition reaction		[100]
Poly (propylene sulfide)120	ROS response	Van der Waals forces	Traumatic brain injury treatment	[101]
4-sulfobenzoic acid attached peptide	Enzyme response	Self-assembly		[102]
Chitosan, hydroxyethyl cellulose, hyaluronic acid	Temperature response	Physical interaction		[103]
Hyaluronic acid and poly vinyl alcohol	Glucose/ROS response	Phenyl boronic ester bonds		[104]
Polyethyleneglycol-polycaprolactone-N1-(4-boronobenzyl)-N3-(4-boronobenzyl)-N1,N1,N3,N3-tetramethylpropane-1,3-diaminium	ROS response	Phenyl boronic ester bonds	Articular cartilage repair and regeneration	[105]
Chitosan	Temperature/pH response	Schiff base linkages and Physical interaction		[106]
Chitosan and chondroitin sulfate	Ultrasound responsive	Schiff base linkages		[107]
Chitosan and silk fibroin	Temperature response	Physical interaction		[108]

**Table 2:** Clinically approved hydrogel products, grouped by their material class and broad indication.

Product	Company/Sponsor Institution	Main Constituent	Indication	Status
Artefill	Suneva Medical, Inc.	Polymethylmethacrylate beads, collagen, and lidocaine	Facial wrinkles and folds	Clinically approved
EUFLEXXA	Ferring Pharmaceuticals Inc.	Hyaluronic acid	Knee osteoarthritis	Clinically approved
INFUSE bone graft	Medtronic Sofamor Danek USA, Inc.	Collagen and recombinant human bone morphogenetic protein-2	Spine, oral-maxillofacial, and orthopedic trauma	Clinically approved
Algisyl-LVR Hydrogel Implant	LoneStar Heart, Inc.	Alginate	Heart failure	Clinically approved
Lgisite M Condress	Smith & Nephew	Alginate collagen	Wound	Clinically approved
Comfeel Plus Contour Dressing	Coloplast Corp	Carboxymethylcellulose	Wound	Clinically approved
Argiform	Research Centre BIOFORM	Polyacrylamide and silver ions	Knee osteoarthritis	Clinical trial
VentriGel	Ventrix, Inc.	Native myocardial extracellular matrix	Myocardial infarction	Clinical trial
BiolineRX	BioLineRx Ltd.	Alginate	Myocardial infarction	Clinical trial
Radiesse	Bioform Medical, Inc.	Hydroxylapatite, carboxymethylcellulose	signs of facial fat loss and volume loss	Clinical trial
Elevess	Anika Therapeutics	Hyaluronic acid, lidocaine	Moderate to severe facial wrinkles and folds	Clinical trial

## Abbreviations

ROS—reactive oxygen species; UCST—upper critical solution temperature; LCST—lower critical solution temperature; PNIPAm—Poly(N-isopropylacrylamide); UV—ultraviolet; O₂—oxide; OH—hydroxyl; RO₂—peroxyl; RO—alkoxyl; HOCl—hypochlorous acid; O₃—ozone; ¹O₂—singlet oxygen; H₂O₂—hydrogen peroxide; Con A—concanavalin A; PBA—phenylboronic acid; AgNPs—silver nanoparticles; AM—amikacin; Nap—naphthalene propylamine; DS—sodium diclofenac; CO_2_—carbon dioxide; CNPs—carbon nanoparticles; AMF—alternating magnetic field; MI—myocardial infarction; MMPs—metalloproteinases; rhCol III—recombinant type III humanized collagen; Cur—curcumin; NO—nitric oxide; I/R—ischemia/reperfusion; CDots—carbon dots; TBI—traumatic brain injury; ECM—extracellular matrix; TM—trimethylolpropane triglycidyl ether; MMPs—matrix metalloproteinases; MMP9—matrix metalloproteinase 9; Anti-NGF—anti-nerve growth factor; hUC—MSCs-human umbilical cord mesenchymal stem cells; EVs—extracellular vesicles; MSCs—mesenchymal stem cells; Exos—exosomes; ICA—icariin; KGN—kartogenin.
